# A Carbon Dioxide Limitation-Inducible Protein, ColA, Supports the Growth of *Synechococcus* sp. PCC 7002

**DOI:** 10.3390/md15120390

**Published:** 2017-12-15

**Authors:** Ginga Shimakawa, Satoru Watanabe, Chikahiro Miyake

**Affiliations:** 1 Department of Biological and Environmental Science, Faculty of Agriculture, Graduate School of Agricultural Science, Kobe University, 1-1 Rokkodai-cho, Nada-ku, Kobe 657-8501, Japan; gshimakawa@stu.kobe-u.ac.jp; 2 Department of Bioscience, Tokyo University of Agriculture, Tokyo 156-8502, Japan; s3watana@nodai.ac.jp

**Keywords:** photosynthesis, CO_2_ limitation, oxidative stress

## Abstract

A limitation in carbon dioxide (CO_2_), which occurs as a result of natural environmental variation, suppresses photosynthesis and has the potential to cause photo-oxidative damage to photosynthetic cells. Oxygenic phototrophs have strategies to alleviate photo-oxidative damage to allow life in present atmospheric CO_2_ conditions. However, the mechanisms for CO_2_ limitation acclimation are diverse among the various oxygenic phototrophs, and many mechanisms remain to be discovered. In this study, we found that the gene encoding a CO_2_ limitation-inducible protein, ColA, is required for the cyanobacterium *Synechococcus* sp. PCC 7002 (S. 7002) to acclimate to limited CO_2_ conditions. An S. 7002 mutant deficient in ColA (Δ*colA*) showed lower chlorophyll content, based on the amount of nitrogen, than that in S. 7002 wild-type (WT) under ambient air but not high CO_2_ conditions. Both thermoluminescence and protein carbonylation detected in the ambient air grown cells indicated that the lack of ColA promotes oxidative stress in S. 7002. Alterations in the photosynthetic O_2_ evolution rate and relative electron transport rate in the short-term response, within an hour, to CO_2_ limitation were the same between the WT and Δ*colA*. Conversely, these photosynthetic parameters were mostly lower in the long-term response of a few days in Δ*colA* than in the WT. These data suggest that ColA is required to sustain photosynthetic activity for living under ambient air in S. 7002. The unique phylogeny of ColA revealed diverse strategies to acclimate to CO_2_ limitation among cyanobacteria.

## 1. Introduction

Oxygenic photosynthesis is the most popular anabolic biological activity on earth. From photon energy, water (H_2_O), and atomospheric carbon dioxide (CO_2_), oxygenic phototrophs synthesize photosynthates, including glycogen, starch, and cellulose. This process involves both light and dark reactions in the photosynthetic electron transport system on the thylakoid membrane and the Calvin-Benson cycle in the stroma in chloroplasts, respectively. In the photosynthetic electron transport system, photon energy excites the reaction center chlorophylls (Chl), P680 and P700 in photosystems (PS) II and I, respectively, to drive photosynthetic linear electron transport. In PSII, H_2_O is oxidized to oxygen (O_2_) by photo-oxidized P680, and electrons derived from P680 are transferred to photo-oxidized P700 in PSI through plastoquinone, cytochrome *b*_6_/*f* complex, and plastocyanin or cytochrome *c*_6_. These processes result in the formation of a proton gradient across the thylakoid membrane to produce ATP with chloroplast ATP synthase. Conversely, electrons originating in P700 are transferred to NADP^+^ via ferredoxin and ferredoxin-NADP^+^ reductase. Subsequently, NADPH and ATP are produced by photosynthetic linear electron transport. In the stroma, ribulose 1,5-bisphosphate (RuBP) carboxylase/oxygenase, also known as Rubisco, catalyzes the carboxylation of RuBP with CO_2_ to drive the Calvin-Benson cycle, producing a variety of sugars with NADPH and ATP as reducing agents.

Oxygenic photosynthesis is often limited by the reaction rate of Rubisco due to CO_2_ limitation, because the partial pressure of CO_2_ in the present atmosphere, which is about 40 Pa, is not sufficient to sustain CO_2_-saturated photosynthetic activity with natural environmental variation [1]. For example, drought stress stimulates stomatal closure to block the diffusion of CO_2_ from the atmosphere into plant leaves [2]. Additionally, in aqueous environments, the diffusion efficiency of CO_2_ is about 10^−4^ times lower than that in the atmosphere, which suggests that algae and aquatic or submerged plants can easily face CO_2_ limitation [3,4]. Limiting CO_2_ suppresses photosynthetic CO_2_ assimilation and causes an excess supply of photon energy, an amount over that needed for photosynthesis, to be delivered to the photosynthetic electron transport system, which may cause photo-oxidative damage to PSII and PSI in oxygenic phototrophs [5,6].

Oxygenic phototrophs have developed diverse strategies to prevent and recover from photo-oxidative damage to allow acclimation to limited CO_2_ environments. For example, various molecular mechanisms function in dissipating excess photon energy in the photosynthetic electron transport system under CO_2_ limitation, including O_2_-dependent alternative electron flow (AEF) [7,8], non-photochemical quenching of Chl fluorescence at PSII [9], and oxidation of P700 [10]. Without these protective mechanisms, excess photon energy is transferred to O_2_ to produce reactive oxygen species (ROS), causing photo-oxidative damage in PSII and PSI [5,6,10]. The methods by which photo-oxidative damage are suppressed differ among various oxygenic phototrophs, and unknown molecular mechanisms remain to be characterized.

We reported on the diverse responses of photosynthesis to CO_2_ limitation in cyanobacteria and eukaryotic algae [1,6,11,12,13,14]. The strategies to acclimate to CO_2_ limitation are diverse, even among cyanobacteria, which are known as the progenitors of oxygenic phototrophs. In the cyanobacterium *Synechocystis* sp. PCC 6803 (S. 6803), flavodiiron proteins (FLV) 2 and 4 are highly expressed in response to limited CO_2_ conditions [5,15], and they maintain high electron transport activity by mediating AEF with O_2_ as the final electron acceptor [1,11], which is essential to protect PSII against photo-oxidative damage [5,16]. Conversely, *Synechococcus elongatus* PCC 7942 (S. 7942) does not have FLV2/4 and shows little AEF in limited CO_2_ [1,11,12]. Similar to S. 7942, the marine species *Synechococcus* sp. PCC 7002 (S. 7002) does not possess FLV2/4. Nevertheless, S. 7002 shows a substantial O_2_-dependent AEF under limited CO_2_ conditions with FLV1 and 3 alone, when the cells are grown under ambient air but not high CO_2_ [6,13], which aligns with the strongly enhanced expression of FLV1/3 genes with CO_2_ limitation in S. 7002 [17]. Although neither S. 6803 nor S. 7942 show any photo-oxidative damage in PSI under CO_2_ limitation, even in the absence of FLVs, S. 7002 cannot oxidize P700 without FLV or it will suffer from photo-oxidative damage in PSI when the available CO_2_ is insufficient [6]. These data suggest that the regulation of photosynthetic electron transport in S. 7002 under CO_2_ limitation is substantially different from that in S. 6803 and S. 7942.

In the present study, we focused on the gene *SYNPCC7002_A2492* that encodes a 20-kDa small membrane-associated protein as a candidate gene related to the unique ability of S. 7002 to acclimate to CO_2_-limited conditions. A global transcriptome analysis of S. 7002 by Bryant’s research group showed that expression of *SYNPCC7002_A2492* is 690-fold higher in cells grown in ambient air than in those grown in high CO_2_, which is the greatest change in all genes, even though the *p*-value did not indicate significance [17]. Here, we studied the gene product of a CO_2_ limitation-associated protein, ColA. To elucidate the physiological significance of ColA in S. 7002, we constructed a ColA knockout mutant (Δ*colA*) and compared the growth, Chl and nitrogen contents, oxidative stress, and photosynthetic parameters with those of the wild type (WT) S. 7002.

## 2. Results and Discussion

### 2.1. Growth of S. 7002 WT and ΔcolA under Ambient Air and High CO_2_

**Figure 1:**
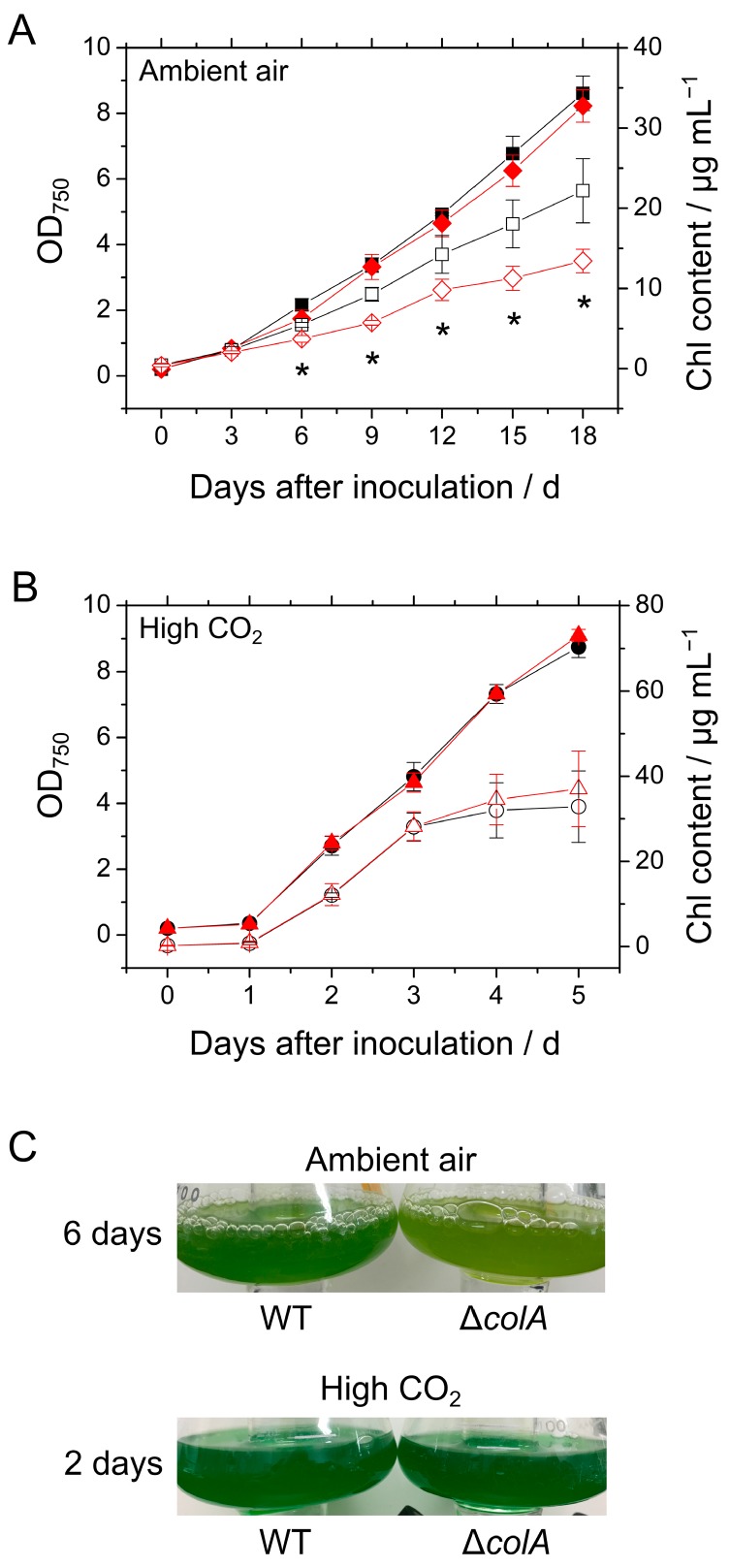
Growth and chlorophyll (Chl) content of the wild type (WT) and Δ*colA* of *Synechococcus* sp. PCC 7002 (S. 7002) under (**A**) ambient air and (**B**) high carbon dioxide (CO_2_). Closed and open symbols, respectively, show the optical density at 750 nm (OD_750_) and Chl content. Black symbols represent S. 7002 WT; red symbols represent, Δ*colA*. Measurements were independently conducted three times, and the data are shown as the mean ± SD. Differences in Chl content between the WT and Δ*colA* were analyzed using Student’s *t*-test. Asterisks indicate statistically significant differences in Chl content between the WT and Δ*colA* at *p* < 0.05. (**C**) Photographs of the culture flasks taken on the indicated days.

To investigate the effects of ColA on the growth of the cyanobacterium S. 7002, we constructed a mutant deficient in the gene *SYNPCC7002_A2492*, Δ*colA* (Supplemental Figure S1), and monitored the growth of S. 7002 WT and Δ*colA* under ambient air and high CO_2_ conditions. Cell growth, reflected in the increase in optical density at 750 nm (OD_750_) of the cultures, was not significantly different between the WT and Δ*colA* in either ambient air or high CO_2_ conditions (Figure 1A,B). However, the Chl content of the Δ*colA* culture was lower than that of the WT under ambient air but not high CO_2_ conditions (Figure 1A,B), which was recognized by the pale color of the Δ*colA* cell culture (Figure 1C). These data suggest that ColA deletion causes a decrease in the amount of Chl in S. 7002 when CO_2_ is limited.

**Figure 2:**
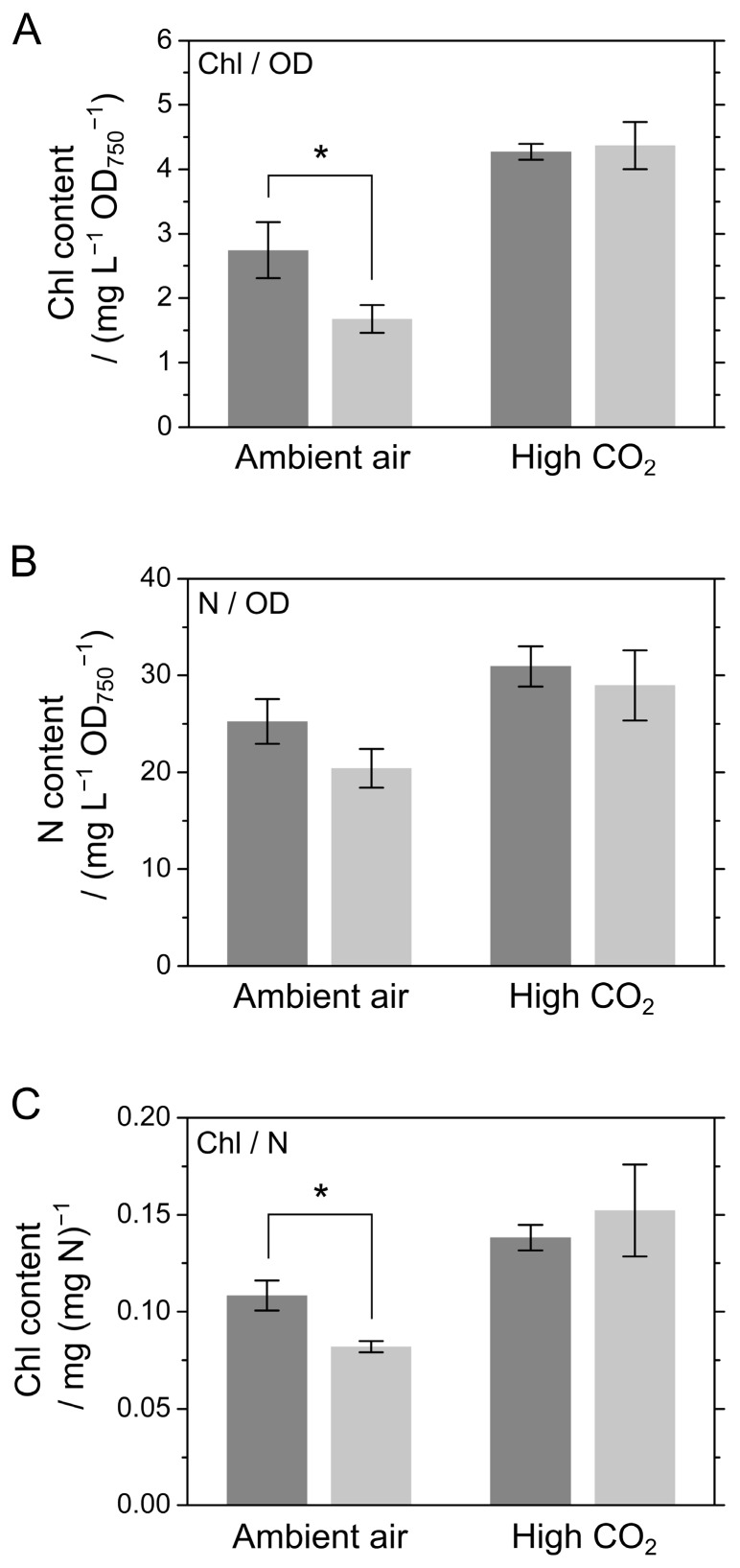
Chlorophyll (Chl) and nitrogen (N) contents of *Synechococcus* sp. PCC 7002 (S. 7002) wild type (WT) and Δ*colA* grown under ambient air and high CO_2_. (**A**) Chl content per optical density at 750 nm (OD_750_). (**B**) N content per OD_750_. (**C**) Chl content per N content. Dark grey bars indicate S. 7002 WT; light grey bars indicate Δ*colA*. Measurements were conducted 8 to 10 and 2 to 3 days after inoculation in cells grown in ambient air and high CO_2_, respectively. Data are shown as the mean ± SD of three independent experiments. Differences between the WT and Δ*colA* were analyzed using Student’s *t*-test. Asterisks indicate statistically significant differences between the WT and Δ*colA* at *p* < 0.05.

Next, we investigated the nitrogen (N) content in S. 7002 WT and Δ*colA*, because in cyanobacteria, a decrease in Chl content can result from a decrease in N content [18]. Therefore, the Chl contents in WT and Δ*colA* were evaluated based on the N content. As shown in Figure 1A, the Chl content per OD_750_ in Δ*colA* was lower than that in the WT under ambient air but not high CO_2_ (Figure 2A). The N content per the OD_750_ in Δ*colA* was generally lower than that in the WT under ambient air, although this difference was not significant (Figure 2B). Furthermore, the Chl content per N in Δ*colA* was significantly lower, by about 70%, than that in the WT in ambient air growth conditions (Figure 2C), which indicates that the lack of ColA impacted the distribution of N under ambient air in S. 7002. Photo-oxidative damage in PSII and PSI is derived from photon energy absorbed by Chl [6,16], which suggests that oxygenic phototrophs can alleviate the potential risk of the photo-oxidative damage by decreasing the Chl content. It is possible that the lower Chl level in Δ*colA* is a secondary effect of the absence of ColA in cyanobacterial cells, allowing it to escape the excess supply of photon energy under CO_2_ limitation conditions. Conversely, in both the WT and Δ*colA*, the Chl and N contents per OD_750_ were likely to be lower in the cells grown in ambient air than in those grown in high CO_2_ (Figure 2). The first step in N assimilation is driven by nitrate reduction catalyzed by nitrate reductase with NAD(P)H as the electron donor, which is reversibly inactivated under CO_2_-limited conditions [19]. This process might support flexible modulation of N use in S. 7002 in response to changes in the amount of available CO_2_.

### 2.2. Oxidative Stress in S. 7002 WT and ΔcolA under Ambient Air

**Figure 3:**
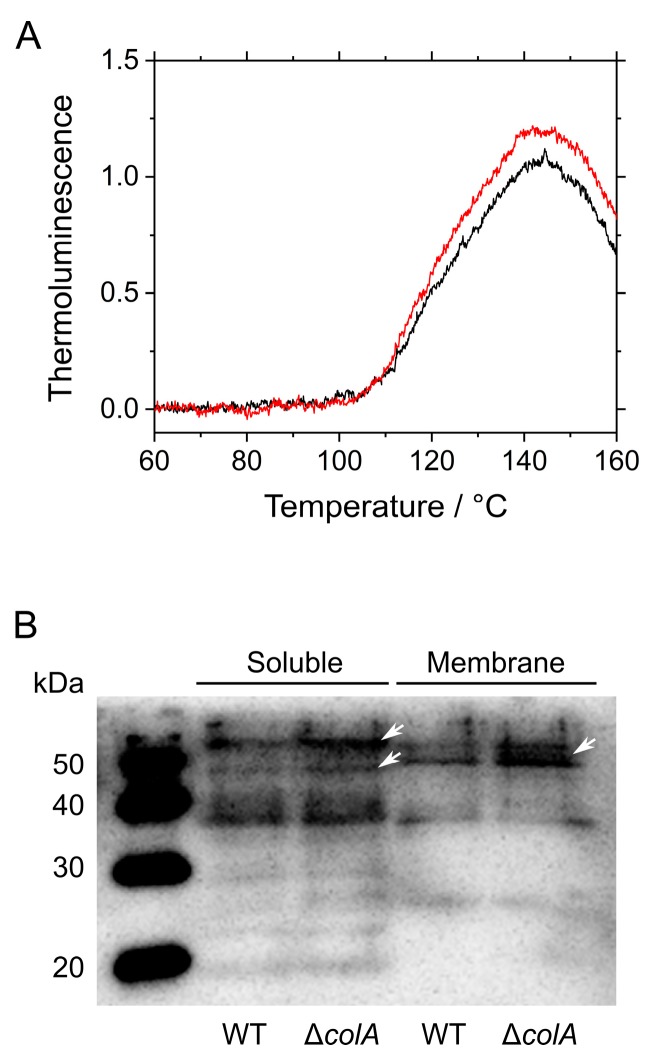
Oxidative stress in *Synechococcus* sp. PCC 7002 (S. 7002) wild-type (WT) and Δ*colA* grown under ambient air. (**A**) High-temperature thermoluminescence signaling related to lipid peroxidation. Black line indicates S. 7002 WT; red line indicates Δ*colA*. Experiments were performed three times, and the average is shown. (**B**) Protein carbonylation detected in crude soluble and membrane fractions. Soluble and membrane fractions extracted from the cells of S. 7002 WT and Δ*colA* (12 and 4 µg protein per lane, respectively) were analyzed by sodium dodecyl sulfate-polyacrylamide gel electrophoresis (SDS-PAGE) and immunoblotting with an antibody specific to 2,4-dinitrophenylhydrazine. We detected an increased signal in Δ*colA* around 50 kDa as indicated by the white arrows.

We evaluated the degree of oxidative stress in the WT and Δ*colA* grown in ambient air. Typical high-temperature thermoluminescence of the cyanobacterial cells reflects the extent of oxidative stress originating from lipid peroxidation in the cells [20]. To measure this stress, cells grown in ambient air were analyzed after adaptation to the dark for 30 min. The luminescence peak, at approximately 140 °C, was higher in Δ*colA* than in the WT (Figure 3A), indicating that lipid peroxidation by ROS was occurring in Δ*colA*.

Next, we quantitatively measured the carbonylated proteins in the WT and Δ*colA* grown in ambient air. Protein carbonylation is caused by sugar- and lipid-derived reactive carbonyls. Sugar-derived reactive carbonyls, including methylglyoxal, glyoxal, and 3-deoxy glucosone, are enzymatically and non-enzymatically produced through sugar metabolism, such as glycolysis and the Calvin-Benson cycle [21,22]. On the other hand, lipid-derived reactive carbonyls, including acrolein, 4-hydroxy-2-nonenal, and malondialdehyde, are produced via lipid peroxidation by ROS [23]. These carbonyls react with amino acid residues, such as lysine and cysteine, to inactivate protein function [24,25]. The amount of carbonylated proteins is an indicator of oxidative stress. Crude soluble and membrane extracts were obtained from cyanobacterial cells grown in ambient air. Protein carbonylation was detected using an antibody specific to 2,4-dinitrophenylhydrazine (DNPH), which conjugates with carbonyl groups. The absence of ColA resulted in an increase in protein carbonylation in both crude soluble and membrane fractions of S. 7002 (Figure 3B). We detected the increased signal particularly around 50 kDa in the crude fractions of Δ*colA*, as indicated by the white arrows in Figure 3B. Cyanobacteria harbor a variety of enzymes that scavenge reactive carbonyls [26,27]. Therefore, the amounts of reactive carbonyls in cyanobacterial cells are determined by both the production and detoxification rates in cells. The impacts of ColA deletion on metabolic alterations of reactive carbonyls should be investigated in future studies.

### 2.3. Responses of Photosynthesis in S. 7002 WT and ΔcolA to CO_2_ Limitation

Based on a lower conversion of N into Chl and the oxidative stress in Δ*colA* under ambient air conditions, ColA was assumed to have a functional role in the suppression of photo-oxidative damage in acclimating to the situations where photosynthetic CO_2_ assimilation is suppressed. To investigate the effects of ColA on photosynthesis in response to CO_2_-limited conditions, we measured the responses of photosynthesis to CO_2_ limitation over both the short- (within an hour) and long-term (a few days) in S. 7002 WT and Δ*colA*.

**Figure 4:**
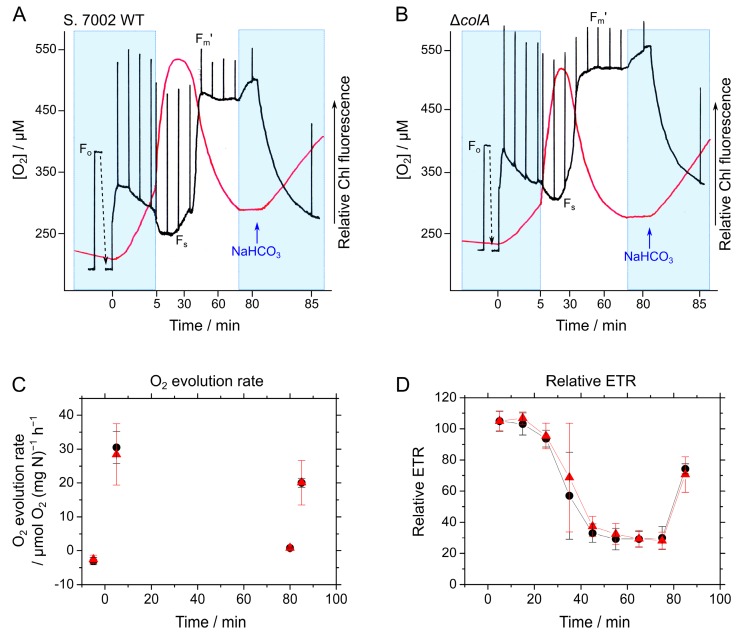
Short-term response of photosynthesis in *Synechococcus* sp. PCC 7002 (S. 7002) wild type (WT) and Δ*colA* to CO_2_ limitation. (**A**,**B**) Time courses of O_2_ in the reaction medium (red lines) and relative chlorophyll (Chl) fluorescence of the cells (black lines) in the WT (**A**) and Δ*colA* (**B**). Red actinic light (AL, 300 μmol photons m^−2^ s^−1^) was turned on at 0 min. Chl fluorescence parameters had the usual definitions (F_o_, minimum fluorescence determined under a measuring light; F_s_, steady-state fluorescence under AL; F_m_′, maximum variable fluorescence under saturating light). NaHCO_3_ (10 mM final concentration) was added as indicated by blue arrows. Blue shading indicates that the top of the O_2_ electrode chamber was closed and that the measurement time scales (*x*-axis) were reduced to 1/10 to determine the O_2_ evolution rate. (**C**,**D**) The O_2_ evolution rate and relative electron transport rate (ETR) were calculated from (**A**,**B**). Black circles, S. 7002 WT; red triangles, Δ*colA*. Measurements were obtained three times independently, and the data are shown as the mean ± SD.

To measure the short-term response, we followed a previously developed method (see the Section 3.8.) [1,11]. Time courses, for both O_2_ in the reaction medium and the relative Chl fluorescence of the cells, were simultaneously measured during the transition of photosynthesis from CO_2_-saturated to CO_2_-limited phases in S. 7002 WT and Δ*colA* grown under high CO_2_ (Figure 4A,B). We calculated both the photosynthetic O_2_ evolution rate (Figure 4C) and relative electron transport rate (ETR) at PSII (Figure 4D) from the data shown in Figure 4A,B. Unexpectedly, no difference was detected in the photosynthetic parameters between the WT and Δ*colA* in the transition to CO_2_-limited conditions (Figure 4). We evaluated the photosynthetic O_2_ evolution rate, based on N content, considering the difference in Chl content between the WT and Δ*colA* (Figure 2). The same photosynthetic responses in Δ*colA* and in WT suggest that ColA does not directly affect photosynthesis and the electron transport reaction. On the other hand, in both the WT and Δ*colA*, the O_2_ evolution rates five minutes after adding sodium bicarbonate (NaHCO_3_) (approximately 20 μmol O_2_ mg^−1^ N h^−1^) were lower than those five minutes after turning on the actinic light (AL) (approximately 30 μmol O_2_ mg^−1^ N h^−1^), corresponding to the relative ETR (Figure 4C,D).

**Figure 5:**
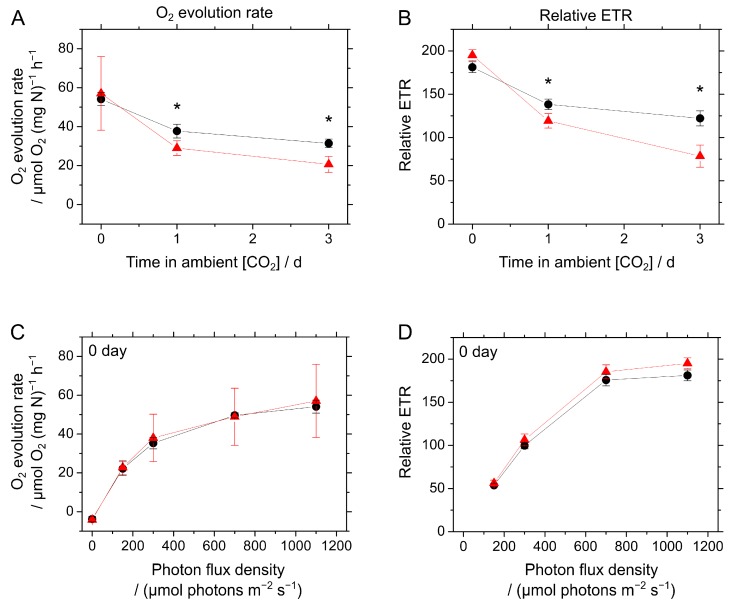

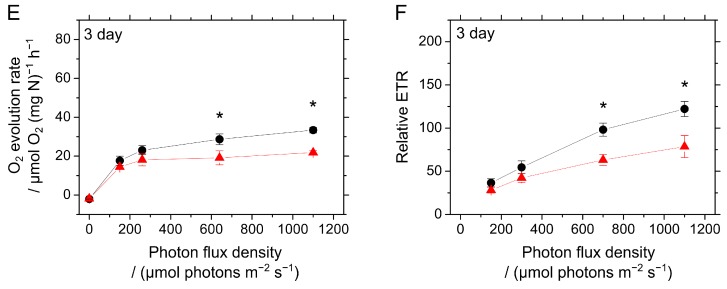
Long-term response of photosynthesis in *Synechococcus* sp. PCC 7002 (S. 7002) wild type (WT) and Δ*colA* to CO_2_ limitation. (**A**,**B**) Time courses of O_2_ evolution rate and relative electron transport rate (ETR) after the cells were shifted from high CO_2_ to ambient air conditions. The photosynthetic O_2_ evolution rate was measured at 1100 µmol photons m^−2^·s^−1^. (**C**−**F**) Dependence of the O_2_ evolution rate and relative ETR on photon flux density (**C**,**D**) before and (**E**,**F**) three days after the transition. Photosynthetic parameters in the reaction medium containing the cells (10 μg Chl mL^−1^ and 10 mM NaHCO_3_) were measured independently three times. Data are shown as the mean ± SD. Black circles, S. 7002 WT; red triangles, Δ*colA*. Differences between the WT and Δ*colA* were analyzed using the Student’s *t*-test. Asterisks indicate statistically significant differences between the WT and Δ*colA* at *p* < 0.05.

Next, we investigated the photosynthesis response to CO_2_-limited conditions on the longer timeframe of a few days. For this measurement, WT and Δ*colA* cells were grown under high CO_2_ conditions, until the OD_750_ reached 2.0 to 3.0 (Figure 1B), and then the cells were inoculated into freshly prepared A^+^ medium with the OD_750_ adjusted to 2.0, and then they were grown in ambient air. We measured both the photosynthetic O_2_ evolution rate and relative ETR before, one day after, and three days after the transition to ambient air growth conditions. In both the WT and Δ*colA*, photosynthetic O_2_ evolution rates and relative ETR gradually decreased with time in ambient air, but the extent of the decrease in photosynthetic parameters was significantly larger in Δ*colA* than in the WT (Figure 5A,B). The dependence of these photosynthetic parameters on photon flux density before and three days after the transition was compared (Figure 5C−F). Although little difference was found in these photosynthetic parameters between the WT and Δ*colA* before the cells were shifted to ambient air (Figure 5C,D), Δ*colA* showed a significantly lower photosynthetic O_2_ evolution rate and relative ETR, particularly at higher photon flux densities, than those in the WT three days after the cells were exposed to ambient air (Figure 5E,F). Unfortunately, we could not determine the reason why photosynthetic activity decreased to a greater extent in Δ*colA*, but the greater oxidative stress in Δ*colA* shown in Figure 3 suggests that the absence of ColA accelerated the photo-oxidative damage and inactivated photosynthetic activity in S. 7002 in ambient air. We assessed the level of total oxidizable P700 (P_m_), which is a marker of PSI activity [6], in the WT and Δ*colA*, which indicated that P_m_ in Δ*colA* (0.52 ± 0.07, *n* = 3) was almost the same as that in the WT (0.53 ± 0.04, *n* = 3), three days after the transition to ambient air growth conditions. In this experiment, we adjusted the concentration of the cyanobacterial cells in the reaction medium to 80 μg N mL^−1^. These data suggest that the larger decrease in photosynthetic activity in Δ*colA* may be due to the inactivation of PSII, rather than of PSI.

### 2.4. Phylogeny of ColA Gene Homologs among Cyanobacteria

**Figure 6:**
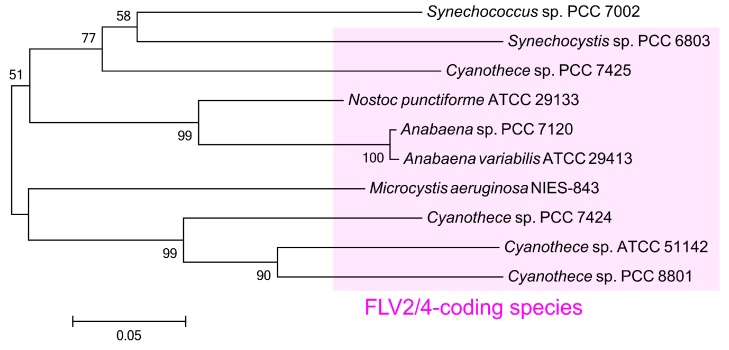
Phylogenetic analysis of the amino acid sequences of ColA in some cyanobacteria. Pink shading shows the cyanobacterial species that harbor flavodiiron protein (FLV) 2 and 4 isozymes.

Gene homologs encoding ColA are conserved in some, but not all, cyanobacteria other than S. 7002. For example, S. 6803 harbors *sll0218* as a ColA gene, whereas S. 7942 has no gene encoding ColA. The gene *sll0218* in S. 6803 is highly expressed under CO_2_-limited conditions, like that observed for *SYNPCC7002_A2492* in S. 7002 [16], and is proposed to stabilize PSII [28]. We aligned the amino acid sequences of ColA isozymes in S. 7002 and S. 6803 (Supplemental Figure S2) and found a 68% similarity between the primary structures of these gene products in the ClustalW analysis. We constructed the phylogenetic tree of ColA isozymes based on amino acid sequences of some cyanobacteria (Figure 6), which indicated that the genes for ColA are normally encoded between the genes for FLV4 and FLV2, designated the *flv4-2* operon, by Aro’ and colleagues, in cyanobacterial genomes [16,28]. In fact, all the cyanobacteria species registered in CyanoBase [29] possess genes for FLV2/4, similar to S. 6803, with S. 7002 as the only exception (Figure 6). Curiously, S. 7002 has a gene for ColA alone, without those for FLV2/4. In S. 6803, the physiological role of ColA encoded by *sll0218* is independent of that of FLV2/4, even though these genes exist in the same operon [28], supporting the idea that ColA functions alone in S. 7002 (Figure 1, Figure 2, Figure 3, Figure 4, Figure 5). The physiological functions of ColA in other FLV2/4-coding cyanobacteria species might provide some insights into the evolutional process of the relationship between ColA and FLV2/4.

We considered the transfer of ColA genes to some cyanobacteria species. In this hypothesis we assumed that the cyanobacteria, like S. 7002, harboring ColA alone diverged, and that ColA had been strongly selected for acclimation to CO_2_-limited conditions. Thereafter, the genes for FLV2/4 were generated by the duplication of genes for FLV1/3 that merged into the location of the gene coding ColA to form the *flv4-2* operon in the genomes of some cyanobacteria, which would have been advantageous for cyanobacteria requiring mechanisms to prevent photo-oxidative damage under CO_2_ limitation conditions. In this study, we constructed a mutant of S. 7942 (#HA-ColA) with the gene for ColA introduced into S. 7002 in the form of a hemagglutinin (HA)-fusion protein (Supplemental Figure S3) and compared the long-term response of photosynthesis to limited CO_2_ to that of a control strain expressing HA alone (#HA) (Supplemental Figure S4). Unfortunately, the heterologous expression of HA-tagged ColA did not affect the response of photosynthesis to limited CO_2_ in S. 7942 (Supplemental Figure S4), implying that another molecular mechanism is responsible for ColA-dependent acclimation of S. 7002 to CO_2_ limitation.

In this study, we found that ColA supports the acclimation of S. 7002 to ambient air growth conditions (Figure 1, Figure 2, Figure 3, Figure 4, Figure 5). ColA gene expression was 690-fold higher in cells grown under ambient air than in those grown under high CO_2_ conditions [17]. Interestingly, gene homologs for ColA are conserved only in a limited number of cyanobacteria, and, even among those cyanobacterial species, their ColA genes are incorporated into the *flv4-2* operon (Figure 6) [28]. That is, S. 7002 is unique in possessing ColA alone. The deletion of ColA impacted the N use (Figure 2), oxidative stress status (Figure 3), and photosynthetic activity (Figure 4 and Figure 5) in ambient air. These data imply that other oxygenic phototrophs without ColA use mechanisms that are different from that of S. 7002 to acclimate to limited CO_2_. We believe that ColA in S. 7002 is evidence of one of the diverse strategies of oxygenic phototrophs used to facilitate cyanobacterial life on earth, and that such a unique strategy for the acclimation to CO_2_ limitation possibly gives us an opportunity to increase a biomass yield for biotechnological applications in the present atmosphere, where the partial pressure of CO_2_ is about 40 Pa.

The latest research [28] has concluded that ColA functions in the process of PSII assembly and repair in S. 6803, which can explain the results in this study. The impairment of the PSII assembly and repair process causes the decreases in Chl and PSII content [28], possibly leading to oxidative stress. Additionally, PSII assembly and repair is a highly coordinated process that requires all components to be carefully controlled, which may be consistent with the failure to complement ColA into S. 7942 (Supplemental Figure S4). In S. 6803, a PSII assembly factor YCF48 is required for the expression of ColA (i.e., Sll0218) [28]. The isozyme for YCF48 is also found in the genome of S. 7002, which is encoded in *SYNPCC7002_A0229* [29]. As described above, the cyanobacterium S. 7002 possesses ColA but not FLV2/4. Therefore, S. 7002 is expected to provide novel insights into the detailed role of ColA, as a distinct component and separate player from that of the FLV2/4 heterodimer, in protection of PSII in future studies.

## 3. Materials and Methods

### 3.1. Growth Conditions and Determination of Chla

Cultures of S. 7002 were kept under continuous light conditions (25 °C, 50 µmol photons m^−2^ s^−1^ from a fluorescent lamp) on A^+^ medium agar plates [30]. For physiological measurements, the cells were inoculated into A^+^ liquid medium and were grown on a rotary shaker (100 rpm) in continuous light (25 °C, 150 μmol photons m^−2^·s^−1^ from a fluorescent lamp) at high CO_2_ (2% (*v*/*v*)) or ambient air. The optical density (OD) of the cultures at 750 nm was measured with a spectrophotometer (U-2800A, Hitachi, Tokyo, Japan).

Cells of the S. 7942 mutants, grown in BG-11 medium rather than A^+^ medium, proliferated as well as S. 7002 [31].

For Chl measurement, cells from 0.1–1.0 mL of culture medium were centrifugally harvested and resuspended by vortexing in 1 mL 100% (*v*/*v*) methanol. After incubation at room temperature for 5 min, the suspensions were centrifuged at 10,000× *g* for 5 min. Total Chl*a* in the supernatants were spectrophotometrically determined [32].

### 3.2. Bioinformatics

All of the cyanobacterial gene sequence data used in this study were obtained from CyanoBase (http://genome.microbedb.jp/CyanoBase) [29].

To construct the phylogenetic tree of gene homologs for ColA, we searched for gene homologs among cyanobacteria using the BLAST tool in CyanoBase. Amino acid sequences of these gene products were aligned in ClustalW [33]. The evolutionary history of these genes was inferred using the neighbor-joining method [34]. The percentages of replicate trees, in which the associated taxa clustered together in the bootstrap test with 500 replicates, are shown next to the branches [35]. The tree was drawn to scale, with branch lengths in the same units as those of the evolutionary distances used to infer the phylogenetic tree. The evolutionary distances were computed using the Poisson correction method [36] and are expressed in the number of amino acid substitutions per site. Evolutionary analyses were conducted in MEGA7 [37].

To predict the transmembrane helices in ColA (Supplemental Figure S2), we used a classification and secondary structure prediction system (SOSUI) [38].

### 3.3. Statistical Analysis

Student’s *t*-tests were applied to detect differences. All statistical analyses were performed using Microsoft Excel 2010 (Microsoft, Washington, DC, USA) and JMP8 (SAS Institute Inc., Tokyo, Japan).

### 3.4. Generation of Mutants

To construct the mutant Δ*colA*, the genomic region encoding *SYNPCC7002_A2492* was amplified from the genomic DNA of S. 7002 by PCR (primer set: forward, CAGCCTCTCCGTCAATGGCTCAA; reverse, CCAGGCGGGATTATTACATGACC) and then cloned into the pGEM-T Easy vector (Promega, Tokyo, Japan). The recombinant plasmid was linearized and amplified by inverse polymerase chain reaction (PCR) (primer set: forward, TGACCGTGTGCTTCTGAAGTGTCGATTGTTTCTGTGCT; reverse, TCTTACGTGCCGATCAACGAAACAGCTCTACCAAAATCAG) and then applied to the In-Fusion cloning system (Takara, Shiga, Japan) with a chloramphenicol resistance gene cassette originating from the pACYC184 vector [11,39]. S. 7002 was transformed with the resulting plasmid using the standard procedure [40]. The mutant was selected on a 0.5% A^+^ medium agar plate containing chloramphenicol (15 µg·mL^−1^), and complete segregation was confirmed by PCR (Supplemental Figure S1).

To introduce the HA-tagged ColA into S. 7942, we used pNSHA based on pNSE1 harboring a spectinomycin resistance gene in the fragment derived from the neutral site [41]. The coding region for ColA was amplified from the genome of S. 7002 and then cloned into pNSHA, digested with *Bam*HI and *Hind*III, using an In-Fusion cloning system (Takara, Shiga, Japan). The resulting plasmid was used to transform WT S. 7942 according to the method used for S. 7002. Heterologous expression of HA-tagged ColA was achieved in the light for 2 h in the presence of isopropyl *β*-d-1-thiogalactopyranoside, which was examined by immunoblotting as described below.

### 3.5. Immunoblot Analysis

For the immunodetection of HA-tagged ColA, cyanobacterial cell cultures (20 mL) were harvested by centrifugation, and the pellets were suspended in 500 µL extraction buffer (50 mM HEPES-KOH, 800 mM sorbitol, 20 mM CaCl_2_, and 1 mM phenylmethylsulfonyl fluoride; pH 7.5). The suspensions were homogenized with glass beads using Bug Crasher GM-01 (Taitec Co., Aichi, Japan) at 4 °C for 20 min and thereafter were centrifuged at 8000× *g* for 15 min at 4 °C. The supernatants were incubated at room temperature for 1 h in Laemmli SDS sample buffer with 6 M urea and then centrifuged at 13,000× *g* for 10 min at 4 °C. The resulting supernatants (10 µL each) were analyzed by sodium dodecyl sulfate-polyacrylamide gel electrophoresis (SDS-PAGE). After electrophoresis, the proteins were electrotransferred to a polyvinylidene fluoride membrane and detected by HA-specific antibody (Aviva Systems Biology, San Diego, CA, USA).

To analyze protein carbonylation, 20 mL cyanobacterial cell cultures were harvested by centrifugation, and the pellets were suspended in a 500 µL extraction buffer (50 mM HEPES-KOH, 800 mM sorbitol, 20 mM CaCl_2_, and 1 mM phenylmethylsulfonyl fluoride; pH 7.5). The suspensions were homogenized with glass beads using Bug Crasher GM-01 (Taitec Co., Aichi, Japan) at 4 °C for 20 min, and thereafter were centrifuged at 8000× *g* for 15 min at 4 °C, resulting in supernatants and pellets. The supernatants were centrifuged twice more at 13,000× *g* for 30 min at 4 °C, and the resulting supernatants were treated as crude soluble fractions. Conversely, the pellets were mildly vortexed in the extraction buffer with 2% (*w*/*v*) octyl *β*-d-glucopyranoside for 30 min at 4 °C. After centrifugation at 13,000× *g* for 30 min at 4 °C, the resulting supernatants were treated as crude membrane fractions. Protein concentrations in both crude fractions were determined using a Pierce 660 nm Protein Assay (Thermo Scientific, Rockford, IL, USA) with bovine serum albumin as the standard. Both crude soluble and membrane fractions (12 and 4 µg protein per lane, respectively) were analyzed by SDS-PAGE. After electrophoresis and electrotransfer to a polyvinylidene fluoride membrane, the proteins were derivatized with 2,4-dinitrophenylhydrazine (DNPH) and then detected by polyclonal antibody specific to DNPH groups [42] purchased from Cosmo Bio (Tokyo, Japan).

### 3.6. Measurement of Nitrogen

Cyanobacterial cells were centrifugally harvested and dried overnight at 60 °C. Dried pellets were digested using the Kjeldahl method with sulfuric acid and hydrogen peroxide (H_2_O_2_). Total nitrogen (N) content was determined using Nessler’s reagent after adding sodium potassium tartrate and sodium hydroxide (NaOH) [18].

### 3.7. Thermoluminescence

Lipid peroxidation was monitored by measuring the in vivo thermoluminescence from 20 °C to 160 °C at a heating rate of 1 °C min^−1^ using a Thermoluminescence System TL 500 (Photon Systems Instruments, Brno, Czech Republic) [43].

### 3.8. Measurement of O_2_ and Chl Fluorescence

Net uptake and evolution of O_2_ were simultaneously measured with Chl fluorescence. Cell samples in a freshly prepared medium (2 mL, 10 μg Chl mL^−1^) were stirred with a magnetic microstirrer and illuminated with red actinic light (AL) (620 < *λ* < 695 nm) at 25 °C. A halogen lamp (Xenophot HLX 64625, Osram, München, Germany) with an LS2 light (Hansatech, King’s Lynn, UK) was used as the AL source. O_2_ was continuously monitored using an O_2_ electrode (Hansatech, King’s Lynn, UK) [1].

The relative Chl fluorescence originating from Chl*a* was measured using a PAM-Chl fluorometer (PAM-101; Walz, Effeltrich, Germany) [1,44]. Pulse-modulated excitation was achieved using an light emitting diode (LED) lamp with a peak emission at 650 nm. Modulated fluorescence was measured at *λ* > 710 nm (Schott RG9 long-pass filter). The minimum Chl fluorescence (*F*_o_) was determined based on illumination using a measuring light (ML). Steady-state fluorescence (*F*_s_) was monitored under AL, and 1000-ms pulses of saturated light (*λ* > 620 nm; 10,000 μmol photons m^−2^·s^−1^) were supplied to determine the maximum variable fluorescence (*F*_m_′). The fluorescence terminology used in this study follows that used in a previous report [45]. The effective quantum yield of PSII, Y(II), was defined as (*F*_m_′–*F*_s_)/*F*_m_′ [46]. The relative electron transport rate (ETR) at PSII was calculated as the product of Y(II) and the photon flux density of AL.

For the S. 7942 mutants, the cells were measured similar to the method used for the S. 7002 cells, with the exception that the reaction medium was replaced with 50 mM HEPES-KOH at pH 7.5.

For the experiments of Figure 4, we simultaneously measured O_2_ and relative Chl fluorescence by the method previously reported [1,11,13]. Cyanobacterial cells, grown in a fresh medium under high CO_2_ conditions, were applied to an O_2_ electrode chamber without an additional inorganic carbon source and were then illuminated with red AL. Illumination with AL stimulated photosynthesis, which was accompanied by an increase in O_2_ in the reaction medium (Figure 4A,B). Thereafter, the CO_2_ in the medium was gradually removed by cyanobacterial photosynthesis, as the diffusion of CO_2_ from the atmosphere into the reaction medium was much slower than its consumption, as a result of photosynthetic CO_2_ assimilation in the experimental system. When the CO_2_ was depleted, O_2_ in the reaction medium began to decrease (Figure 4A,B), indicating that photosynthesis was suppressed in the transition to CO_2_-limited conditions. The addition of CO_2_, in the form of NaHCO_3_ to the reaction medium, restored photosynthetic activity (Figure 4). During measurement, the top of the chamber remained open, which enabled O_2_ and CO_2_ to diffuse into or out of the reaction medium. This open system prevented an excessive increase in O_2_ in the reaction mixture during the longer measurements, so that the level did not surpass the undetectable point of the O_2_ electrode. We temporarily closed the chamber to exclude the effects of O_2_ diffusion to determine the photosynthetic O_2_ evolution rate as indicated by blue shadings (Figure 4A,B). With this experimental system, we could evaluate the response of photosynthesis to CO_2_ limitation in vivo [1,11,13].

### 3.9. Measurement of Total Oxidizable P700

Total oxidizable P700 (P_m_) of the cyanobacterial cell samples was measured using a Dual-PAM-100 (Heintz Walz, Effeltrich, Germany) at room temperature (25 ± 2 °C). The reaction mixture consisted of fresh A^+^ medium and cells (10 μg Chl mL^−1^). The maximum P700 photo-oxidation level, obtained with saturated pulse light under far-red illumination, was measured and defined as P_m_ according to the standard method [47]. For measurements, a 300-ms saturation pulse (10,000 µmol photons m^−2^·s^−1^) was supplied.

## Supplementary Materials

The following are available online at www.mdpi.com/1660-3397/15/12/390/s1, Figure S1: DNA fragments amplified by polymerase chain reaction (PCR) showing complete segregation of the inactivated gene *SYNPCC7002_A2492*; Figure S2: Comparison of the amino acid sequences of ColA in *Synechococcus* sp. PCC 7002 and *Synechocystis* sp. PCC 6803; Figure S3: Immunoblotting to detect heterologous expression of ColA in *Synechococcus elongatus* PCC 7942; Figure S4: Effects of heterologous expression of ColA on the long-term response of photosynthesis in *Synechococcus elongatus* PCC 7942 to limited CO_2_.
