# Supplementary material for: A Carbon Dioxide Limitation-Inducible Protein, ColA, Supports the Growth of Synechococcus sp. PCC 7002
Source: Mar Drugs. 2017 Dec 15;15(12):390. doi: 10.3390/md15120390 (PMC5742850; doi:10.3390/md15120390)
Supplement: Supplementary file 1 [file marinedrugs-15-00390-s001.zip › Figures_Supplements.pptx]

## Slide 1
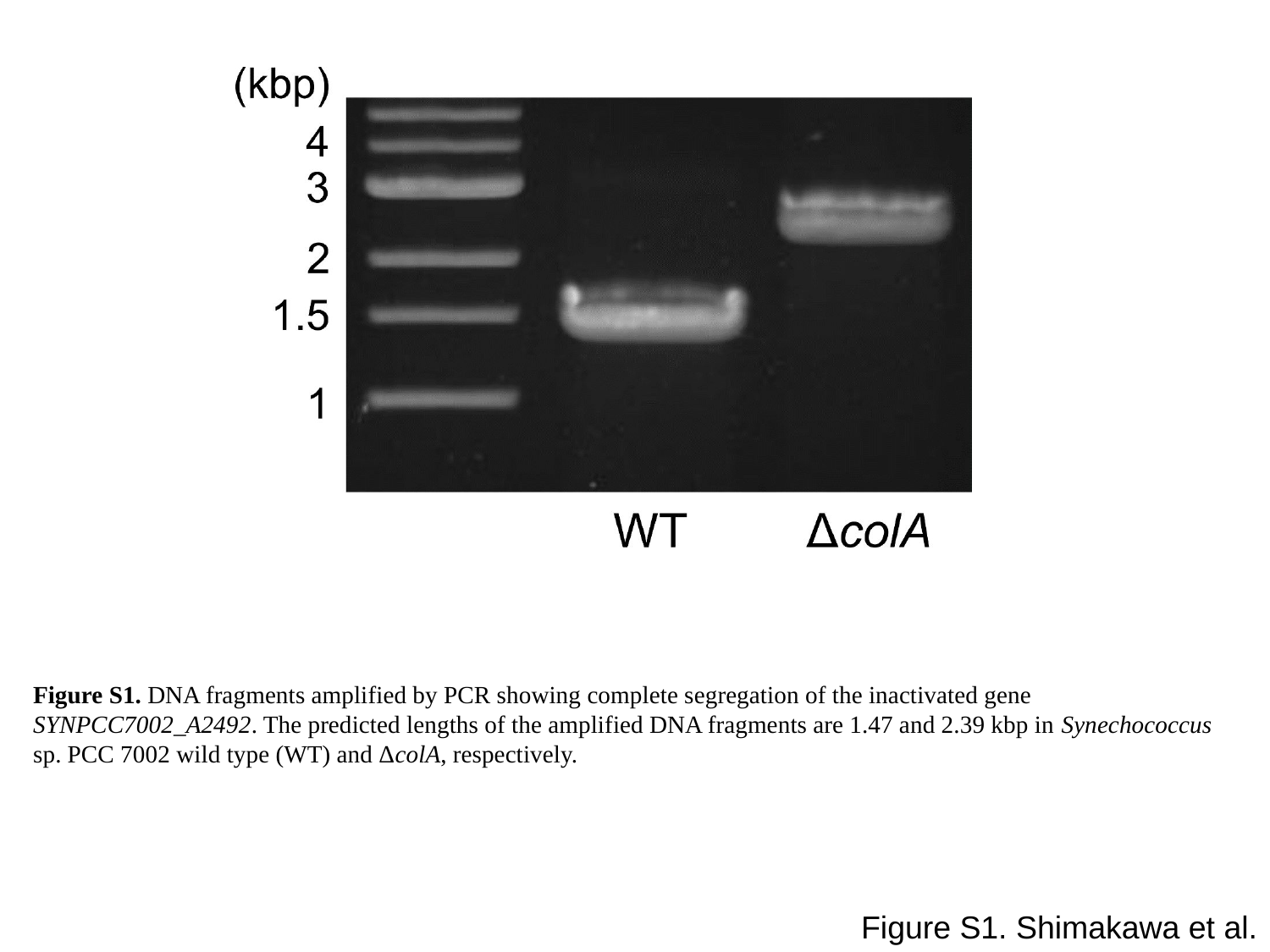

Figure S1. DNA fragments amplified by PCR showing complete segregation of the inactivated gene SYNPCC7002_A2492. The predicted lengths of the amplified DNA fragments are 1.47 and 2.39 kbp in Synechococcus sp. PCC 7002 wild type (WT) and ΔcolA, respectively.
Figure S1. Shimakawa et al.

## Slide 2
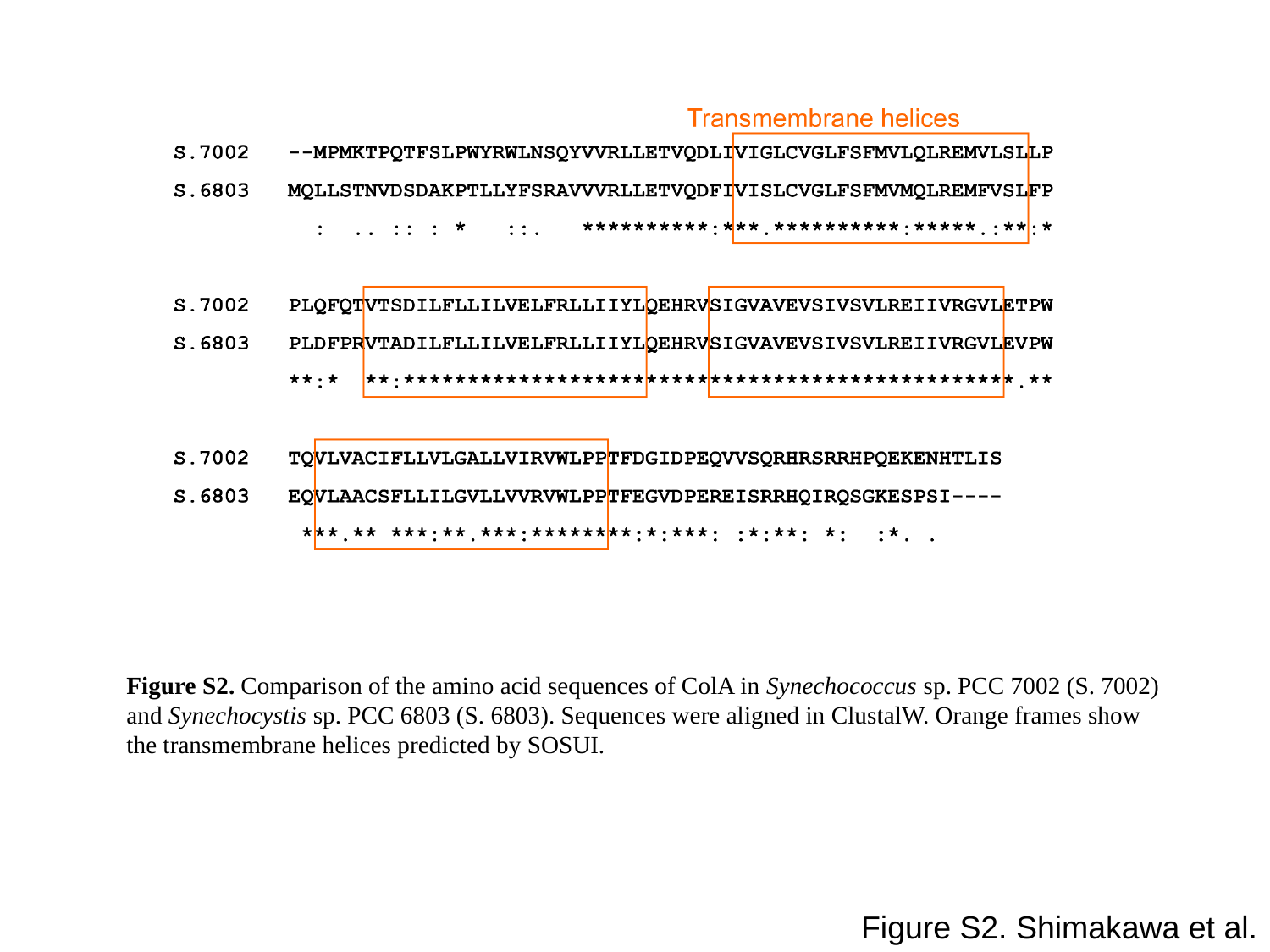

Figure S2. Comparison of the amino acid sequences of ColA in Synechococcus sp. PCC 7002 (S. 7002) and Synechocystis sp. PCC 6803 (S. 6803). Sequences were aligned in ClustalW. Orange frames show the transmembrane helices predicted by SOSUI.
Figure S2. Shimakawa et al.

## Slide 3
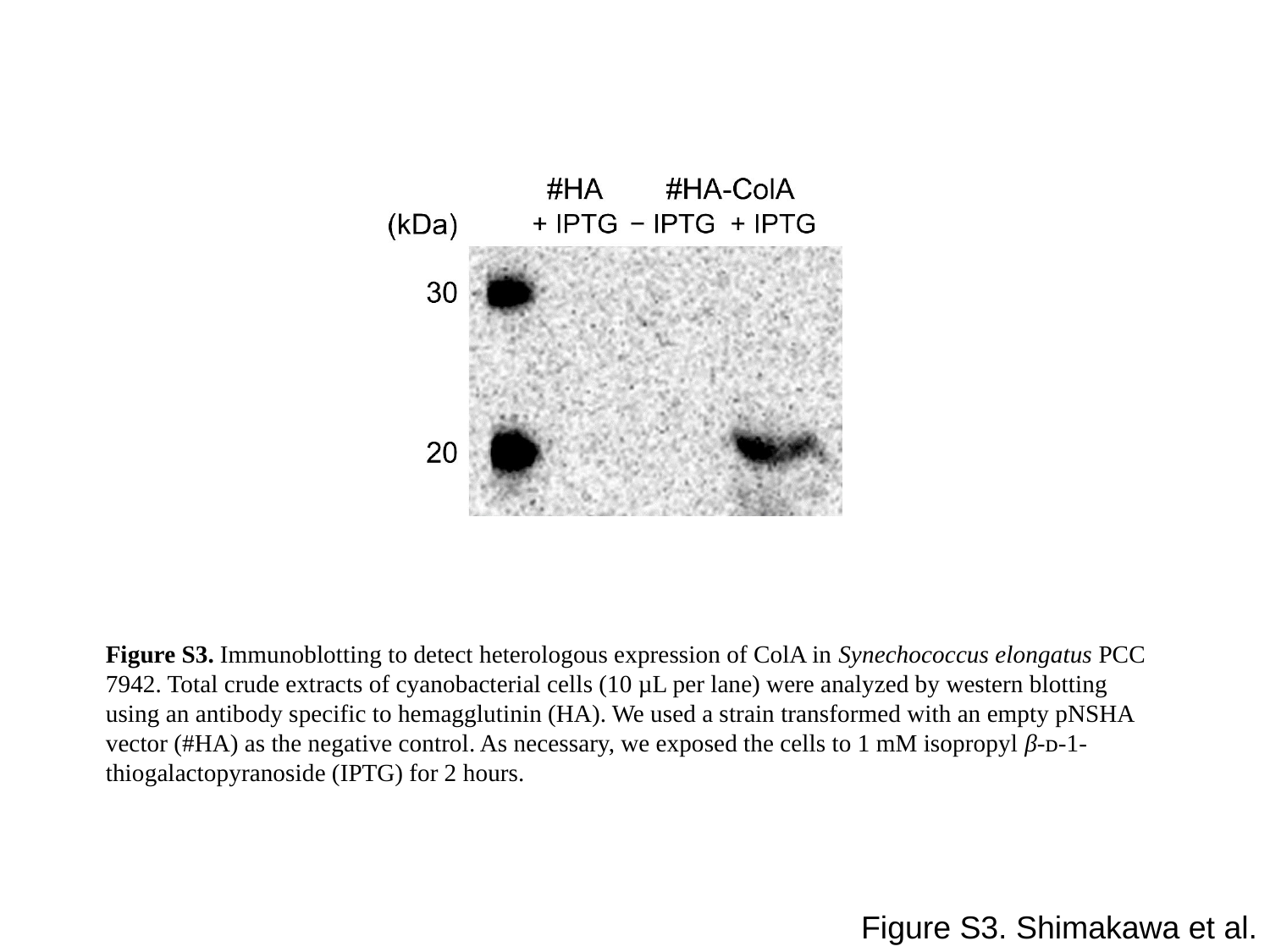

Figure S3. Immunoblotting to detect heterologous expression of ColA in Synechococcus elongatus PCC 7942. Total crude extracts of cyanobacterial cells (10 µL per lane) were analyzed by western blotting using an antibody specific to hemagglutinin (HA). We used a strain transformed with an empty pNSHA vector (#HA) as the negative control. As necessary, we exposed the cells to 1 mM isopropyl β-ᴅ-1-thiogalactopyranoside (IPTG) for 2 hours.
Figure S3. Shimakawa et al.

## Slide 4
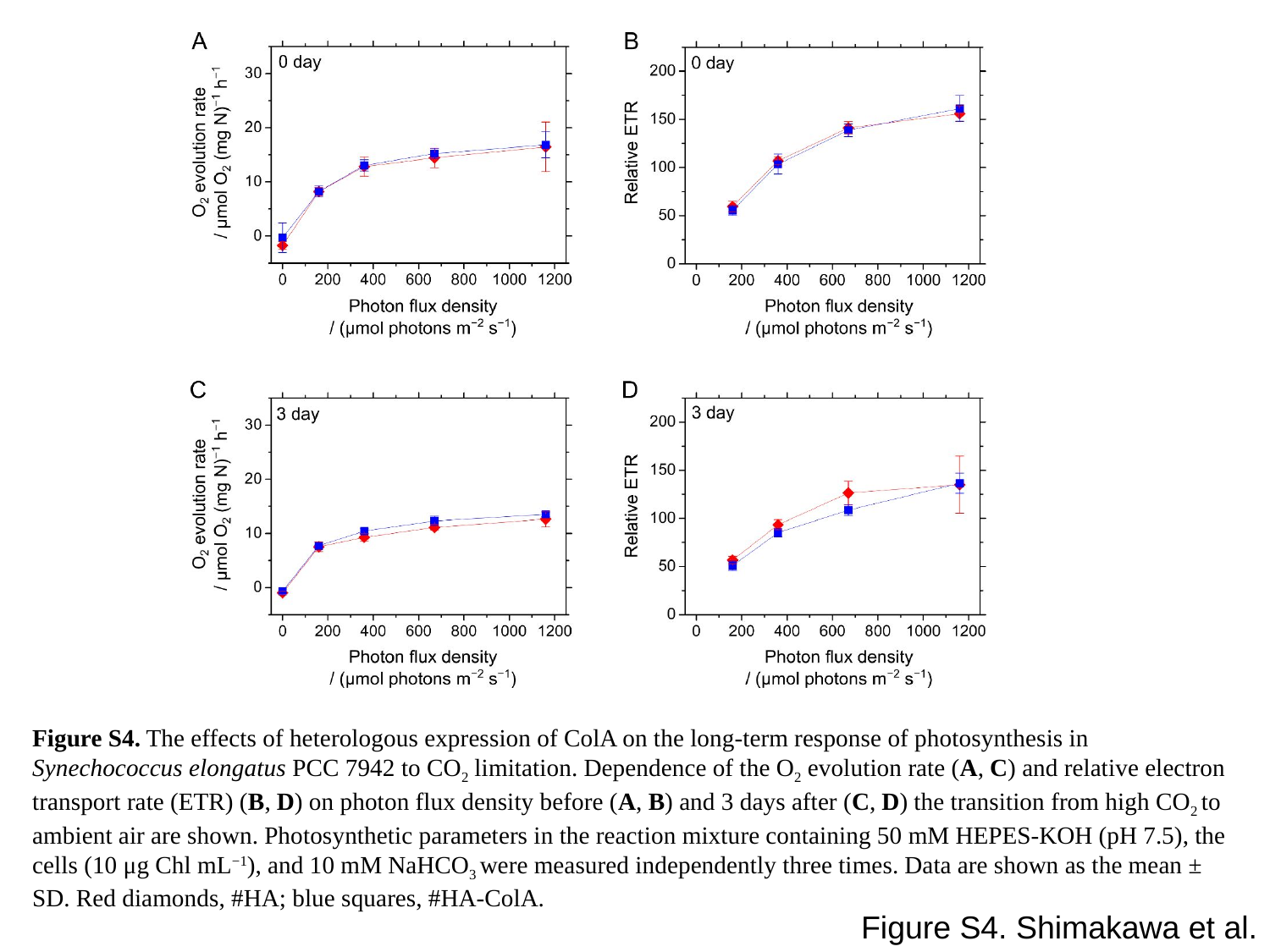

Figure S4. The effects of heterologous expression of ColA on the long-term response of photosynthesis in Synechococcus elongatus PCC 7942 to CO2 limitation. Dependence of the O2 evolution rate (A, C) and relative electron transport rate (ETR) (B, D) on photon flux density before (A, B) and 3 days after (C, D) the transition from high CO2 to ambient air are shown. Photosynthetic parameters in the reaction mixture containing 50 mM HEPES-KOH (pH 7.5), the cells (10 μg Chl mL−1), and 10 mM NaHCO3 were measured independently three times. Data are shown as the mean ± SD. Red diamonds, #HA; blue squares, #HA-ColA.
Figure S4. Shimakawa et al.
